# Dynamic changes of cytotoxic T lymphocytes (CTLs), natural killer (NK) cells, and natural killer T (NKT) cells in patients with acute hepatitis B infection

**DOI:** 10.1186/1743-422X-8-199

**Published:** 2011-05-02

**Authors:** Jun Li, Yaping Han, Ke Jin, Yufeng Wan, Shixia Wang, Bo Liu, Yuan Liu, Shan Lu, Zuhu Huang

**Affiliations:** 1 Department of Infectious Diseases, The First Affiliated Hospital with Nanjing Medical University, Nanjing 210029, China; 2 China-US Vaccine Research Center, The First Affiliated Hospital with Nanjing Medical University, Nanjing 210029, China; 3 Laboratory of Nucleic Acid Vaccines, Department of Medicine, University of Massachusetts Medical School, Worcester, MA 01605, USA

## Abstract

****Background**:**

The goal of this study is to observe changes in HBcAg-specific cytotoxic T lymphocytes (CTLs), natural killer (NK) and natural killer T (NKT) cells from peripheral blood and to relate such changes on viral clearance and liver injury in patients with acute hepatitis B (AHB).

****Methods**:**

Dynamic profiles on the frequency of HLA-A0201-restricted HBcAg18-27 pentamer complex (MHC-Pentamer)-specific CTLs and lymphocyte subsets in AHB patients were analyzed in addition to liver function tests, HBV serological markers, and HBV DNA levels. ELISPOT was used to detect interferon-gamma (INF-γ) secretion in specific CTLs stimulated with known T cell epitope peptides associated with HBV surface protein, polymerase, and core protein.

****Results**:**

HBV-specific CTL frequencies in AHB patients were much higher than in patients with chronic hepatitis B (CHB) (p < 0.05). HBeAg and HBV DNA disappeared earlier in AHB patients with a high frequency of HBV-specific CTLs compared with those with a low frequency of HBV-specific CTLs (p = 0.001 and 0.024, respectively). INF-γ spots of effector cells stimulated by Pol575-583, Env348-357, or Core18-27 epitope peptides were significantly greater in AHB patients than in CHB patients (p < 0.01). CD3^+^CD8^+ ^T cell numbers in AHB patients was more than observed in the healthy control group from the first to the fourth week after admission (p *= *0.008 and 0.01, respectively); the number of CD3^+^CD8^+ ^T cells and frequency of HBcAg18-27-specific CTLs in AHB patients reached peak levels at the second week after admission. NK and NKT cell numbers were negatively correlated with the frequency of HBcAg-specific CTLs (*r *= -0.266, p = 0.05).

****Conclusions**:**

Patients with AHB possess a higher frequency of HBcAg-specific CTLs than CHB patients. The frequency of specific CTLs in AHB patients is correlated with HBeAg clearance indicating that HBV-specific CTLs play an important role in viral clearance and the self-limited process of the disease. Furthermore, NK and NKT cells are likely involved in the early, non-specific immune response to clear the virus.

## Background

The clinical manifestations and outcomes of hepatitis B virus (HBV) infection depend mainly on the intensity and type of anti-viral immunity produced by the infected individual. In patients with acute HBV infection, the specific immune response induced against HBV is a strong, polyclonal, multi-specific cellular immune response, and the virus is eventually cleared. However, in patients with chronic hepatitis B, the specific cellular immune response to HBV is weak and the body is immune-tolerant to HBV. Previous studies have shown that the roles of immune cells in the anti-viral immune response are not independent [1] and instead, cooperation among different subsets of immune cells may exist. In the current study, we analyzed the frequency and functional changes of specific cytotoxic T lymphocytes (CTLs) in AHB patients and the possible dynamic relationship between lymphocyte subsets and the number of NK and NKT cells. We also explored the roles of specific and non-specific immune cells in viral clearance and cell injury. The results from these studies could provide us with the means to evaluate the clinical prognosis of patients with hepatitis B and to develop prevention and control strategies.

In the current report, we show that patients with AHB possess a higher frequency of HBcAg-specific CTLs than CHB patients and the frequency of specific CTLs in AHB patients is correlated with HBeAg clearance indicating that HBV-specific CTLs play an important role in viral clearance and the self-limited process of the disease.

## Methods

### Subjects

This study included 131 inpatients and outpatients with HBV infection from the Infectious Diseases Department of Jiangsu Province Hospital from August 2006 to July 2010. The clinical features of these patients are shown in Table 1. The subjects were classified according to HLA genotype. HLA-A0201-positive patients made up the HBV-specific CTLs test group and HLA-A0201-negative patients made up the specific antigen epitope control group. The diagnosis of AHB was based on typical clinical manifestations, i.e., increased alanine transaminase (ALT) to at least 2.5 times the normal upper limit, no history of hepatitis B, a positive test for hepatitis B surface antigen (HBsAg) and serum anti-HBc IgM [2], ALT returning to normal and the disappearance of HBsAg, and HBeAg within six months after the onset of illness. The diagnosis of CHB was based on the guidelines for chronic hepatitis B diagnosis of the American Association for the Study of Liver Diseases (AASLD) [3]. Patients who had an infection with other hepatitis viruses, a history of autoimmune disease, a history of hepatotoxic drug use or a history of nucleoside anti-HBV drug or interferon use were excluded. A total of 36 healthy volunteers with normal liver function, negative HBV serological markers, and no history of hepatitis A virus (HAV), hepatitis C virus (HCV), hepatitis D virus (HDV), hepatitis E virus (HEV), cytomegalovirus, or Epstein Barr virus (EBV) infection were used as a negative control group for the detection of specific CTLs. The current study was reviewed and approved by the Institutional Ethics Committee and consents were received from patients included in the current study.

**Table 1:** General information about the study subjects

Variant		Case (131)		Normal (50)
			
		AHB (53)	CHB (78)	
Sex	Male	38	51	33
	
	Female	15	27	17

Age		18-64 (40.2)	20-53 (31.5)	20-52 (27.5)

HLA-A0201	Positive	18	21	10
	
	Gene frequency (%)	33.96	26.9	20.0

Transmission route	Sexual	4	0	NA
	
	Vertical	0	21	NA
	
	Injection	2	0	NA
	
	Nonspecific reason	47	57	NA

### Major reagents

A genomic DNA mini-spin kit was purchased from QIAGEN (Cat. No. 12143). Reverse sequence-specific oligonucleotide probes (R-SSO) and genotyping reagents were provided by One Lambda Inc. (R-SSO-008N, CA, USA). Sequence-specific primers (polymerase chain reaction-sequence specific primer, PCR-SSP) and HLA high-resolution typing kits were purchased from Invitrogen (U.S. Batch: 27463). The PCR thermocycler (PCT-200TM) was purchased from M.J Company (USA). The gel imaging analysis system was purchased from Spring (Transilluminator 202D, Cold Spring Harbor, USA). The HBV DNA quantitative detection kit was purchased from PiJi (Shenzhen), and the HBV DNA frequency detector was a LightCycler 1.0 purchased from Roche (USA). The HBcAg18-27 MHC/Pentamer-PE was constructed by Proimmune (U.K.). The HLA-A0201-limitted epitope peptides Corel8-27 (FLPSDFFPSV), Pol575-583 (FLLSLGIHL), Env348-357 (GLSPTVWISV), and HCV Core132-140 (DLMGYIPLV) were synthesized by GL Biochem Ltd (Shanghai). ELISPOT kit was purchased from U-Cytech (Netherlands). The mouse-anti-human CD3-PerCP-Cy5.5 and four-color lymphocyte subset detection kits were purchased from BD Biosciences (USA). The mouse anti-human CD8-APC was purchased from Beckman Coulter Corp. (USA). HBV serological markers detection kit and instrument (Axsym 1.0) were from Abbott (USA). Automatic Biochemical Detector (AU5400) and associated test kits were from Olympus (Japan).

### Analysis of the HLA-A2 allele using high-resolution PCR-SSP

Analysis of the HLA-A2 allele was performed as described elsewhere [4]. Briefly, genomic DNA was extracted from 200 μl of sodium citrate-anticoagulated venous blood. DNA, at a concentration of >50 ng/μl and OD260/280 between 1.7-2.0, was used as the template for HLA-A2 allele analysis. Total reaction volume was 23 μl, including 2 μl of 2.5 pmol/μl forward primer, 2 μl of 2.5 pmol/μl reverse primer, 0.5 μl of 5 U/μl Hot-start Taq enzyme, 2 μl of 10*Buffer, 0.5 μl of 10 mMol dNTPs, 6 μl of ddH2O; the reaction conditions of PCR were 96°C, 1 min, 1 cycle; 96°C for 25 s, 70°C for 50 s, 72°C for 45 s, 5 cycles; 96°C for 25 s, 65°C for 50 s, 72°C for 45 s, 21 cycles; 96°C for 25 s, 55°C for 60 s, 72°C for 120 s, 4 cycles. Electrophoresis of the amplified products was done in a 2% agarose gel stained with ethidium bromide (EB); bands were observed under the UV lamp with a wavelength of 254 nm and UniMatc software was used to compare with the standard map to determine the genotype. A gel imaging analysis system was used for detection and analysis. High-resolution results were obtained with Unityper analysis software. Patients with HLA-A0201 genotype were included into the HBV-specific group.

### Detection of HBcAg-specific CTLs

Detection of HBcAg-specific CTLs was done as previously reported [4]. Briefly, 10 μl HLA-A0201 restricted epitope HBcAg18-27 MHC/Pentamer-PE (amino acid sequence was FLPSDFFPSV) and 100 μl 1 × 10E6 cells/ml peripheral blood lymphocytes were added into tubes special for flow cytometry. The tubes were placed into 4°C in the dark for 20 min, and centrifuged at 500 g for 5 min. The supernatant was discarded, the cells were washed using 3 ml pH7.2 PBS containing 0.1% BSA, the cells were resuspended at 1000 r/min for 30 s, and 20 μl anti-CD3 monoclonal antibody labeled with PerCP Cy5.5 and 20 μl anti-CD8 monoclonal antibody labeled with APC were added. The tubes were placed in the dark at 4°C for 20 min, and were washed then fixed using 500 μl 1% paraformaldehyde. FACS was used for quantitative analysis of the percentage of HBcAg18-27 MHC-Pentamer-PE/CD8-APC double-positive cells with CD3 gating.

### Detection of epitope peptide specific CTLs

Detection of epitope peptide-specific CTLs was performed as described elsewhere [5]. The 96-well polyvinylidene plates were pre-coated with 2 μg/ml of mouse-anti-human interferon gamma (IFN-γ) mAb and incubated at 4°C overnight, washed six times with sterile PBS containing 1% FCS. 100 μl PBMC suspension was added into the wells to ensure 2 × 10E5 cells per well. 100 μl serum-free medium was added to blank control wells; 10 μl PHA at a final concentration of 4 mg/L was added to the positive control wells, which contained 2 × 10E4 cells per well; HLA-A0201-restricted epitope at a final concentration of 10 μg/ml was added to the detection wells; HLA-A0201-restricted HCV Core132-140 (DLMGYIPLV) at a final concentration of 10 μg/ml was added to the negative control wells. Plates were incubated in 5% CO2 at 37°C for 24 h. The medium and cells were removed and 200 μl deionized water was added and incubated on ice for 10 min. After washing ten times with PBS containing 0.05% Tween-20, 100 μl biotinylated anti-IFN-γ antibody was added to each well and plates were incubated at 37°C for 1 hour. The plates were washed again and incubated with HRP-labeled streptavidin at 37°C for 1 h. After washing the plates again, 100 μl of AEC solution was added to each well and incubated at room temperature for 30 min. The color reaction was stopped by washing plates with deionized water. An ELISPOT automatic analysis system was used to count the spots; each spot represented an IFN-γ-secreting cell and the average of the three wells was taken as the detecting value. The number of epitope peptide-specific CTLs was determined as the spots per each epitope peptide group minus the spots in the negative control group.

### Detection of peripheral blood lymphocyte subsets

The cells were marked as previously reported [6] and gated for leukocyte common antigen (CD45)-positive cells. Lymphocyte surface markers were then analyzed by two parameters according to a forward and side scatter two-parameter point diagram. The results were analyzed using CellQuest (BD, USA) software.

### HBsAg, HBeAg and HBV-DNA detection

HBV HBsAg and HBeAg levels were determined using the Architect system (Abbott, HBsAg and HBeAg chemiluminescent microparticle immunoassay kit). The levels of HBV DNA were detected using a LightCycler 1.0.

### Blood biochemistry

ALT, AST, and total bilirubin (TBIL) levels were detected using automatic Biochemical Detector (AU5400) and associated test kits from Olympus, Japan.

### Statistical analysis

The experimental data were analyzed using SPSS 13.0 software. Quantitative data were described using "mean ± standard deviation". Comparisons between groups were analyzed using variance or nonparametric tests (Kruskal-Wallis and Mann-Whitney U tests). The relationship between the two quantitative indicators was analyzed using Pearson correlation analysis. p < 0.05 was considered significant.

## Results

### HLA allele test

High-resolution SSP-PCR results showed that 18 out of 53 AHB patients were HLA-A0201 positive (gene frequency was 33.96%), 21 out of 78 CHB patients were HLA-A0201 positive (gene frequency was 26.9%), and 10 out of 50 healthy controls were HLA-A0201 positive (gene frequency was 20.0%). Although the frequencies of HLA-A0201 positive cases in AHB and CHB were higher than those in healthy controls, this difference was not significant.

### Differences in specific CTLs in different HBV infected patient groups

**Figure 1:**
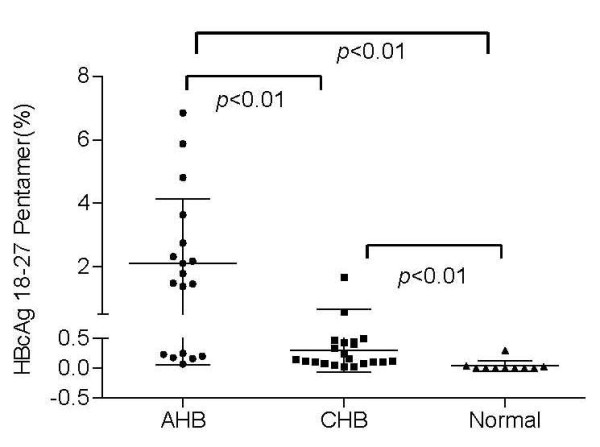
**Frequency of HBcAg 18-27 epitope-specific CTLs**. AHB indicated that the first HBcAg18-27 Pentamer test result after admission among the HLA-A0201-positive patients with acute hepatitis B; CHB indicated that the HBcAg18-27 Pentamer test result among the HLA-A0201-positive patients with chronic hepatitis B; Normal indicated that the HBcAg18-27 Pentamer test result among the HLA-A0201-positive subjects in the healthy control group. The differences among these groups were significant, p = 0.0 01.

Thirty-nine HLA-A0201-positive patients were included in the HBcAg-specific CTLs test group. Among these patients, there were 18 AHB patients and the frequencies of HBcAg-specific CTLs were 1.64 (0.22~2.97), 0.97 (0.35~4.59), 1.05 (0.47~2.34), 0.43 (0.31~1.05), 0.38 (0.24~0.61), and 0.25 (0.16~0.33) from the first to the sixth week after admission, respectively. CTLs frequencies during the first, second, and third week were significantly higher than those from 21 CHB patients, Z = -4.226, -3.804, and -2.537, respectively, (p < 0.05) (Figure 1). In addition, the frequency of HBcAg-specific CTLs in 10 healthy controls was 0.00 (0.00~0.03), which was significantly lower than observed in AHB and CHB patients (p < 0.01).

### Dynamic changes in liver injury and HBV serological markers in AHB patients

The first evaluation of liver function was carried out when AHB patients were admitted; serum HBV DNA, HBV markers, ALT, AST, and TBIL were then detected every week for a total of six measurements, except for HBV DNA quantification and HBV serological marker detection, which continued until they turned negative. HBsAg and HBV DNA were undetectable within three months in all AHB patients. Our results showed ALT, AST, and TBIL in AHB patients were at peak levels upon admission, and had returned to normal in most patients by six weeks. Among the 53 AHB patients, there were 14 patients (26.4%) with undetectable serum HBV DNA upon admission, nine patients turned negative at the second week after admission, 12 at the third week, six at the fourth week, six at the fifth week after admission, and the remaining patients turned negative at the sixth week. Also, among the 53 AHB patients, there were 27 patients (50.9%) with negative serum HBeAg upon admission, five patients turned negative at the second week after admission, eight at the third week, four patients at the fourth week, one patients at the fifth week, and eight at the sixth week. Among these same patients, there were nine patients (17.0%) with negative serum HBsAg upon admission, nine patients turned negative at the second week after admission, 14 patients at the third week, two patients at the fourth week, four patients at the fifth week, seven patients at the sixth week, and the remaining patients turned negative during the period from seventh to eleventh week.

### The relationship between dynamic changes in HBcAg-specific CTLs frequency, liver injury, and viral clearance

**Figure 2:**
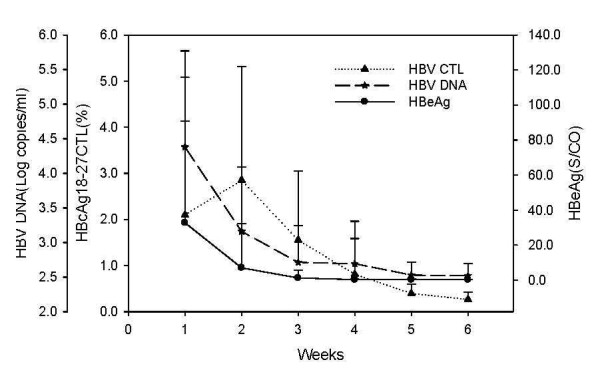
**The relationship between the frequency of peripheral blood HBcAg18-27-specific CTLs and HBV clearance in AHB patients**. HBV infection in AHB patients was measured by the viral load (HBV DNA amount) and level of HBeAg at weeks after admission to hospital.

**Figure 3:**
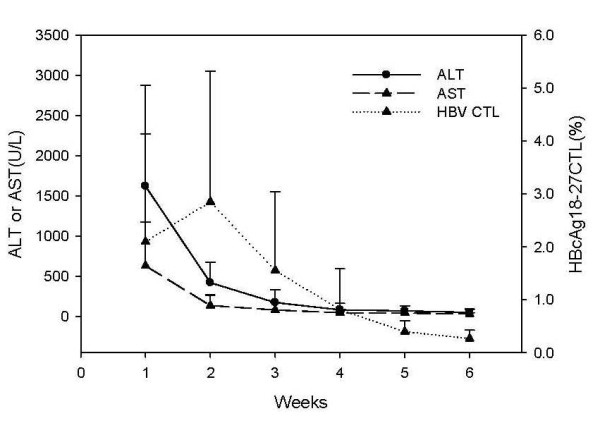
**Relationship between the frequency of peripheral blood HBcAg18-27-specific CTLs and liver injury in AHB patients**. Liver function tests (ALT and AST) were measured in hospitalized AHB patients.

HBcAg-specific CTLs levels among the 18 HLA-A0201-positive AHB patients were tested every week following admission for a total of six measurements (the test upon admission was designated as the first week). Peak HBcAg-specific CTLs in AHB patients occurred at the second week after admission, started to decline at the third week, and declined to the lowest level at the fifth week. With an increase in the frequency of HBcAg-specific CTL, serum HBV DNA declined rapidly, the level of serum HBsAg and HBeAg showed the same tendency, and ALT and other live function indictors also returned to normal (Figure 2 and Figure 3). After the HBV DNA and HBeAg turned negative, HBV-specific CTL stayed at a low level. Among the 18 AHB patients, 11 patients had HBcAg-specific CTLs frequencies greater than 1.0, while seven patients had frequencies < 1.0. The disappearance of HBeAg and HBV-DNA in the patients whose CTLs frequency were greater than 1.0 occurred earlier than that in the patients whose CTLs frequency were less than 1.0, t = -4.313 and -2.485, respectively (p *= *0.001 and 0.024).

### Function Comparison between the CTLs of AHB patients who were in acute phase and the CTLs of CHB patients

**Figure 4:**
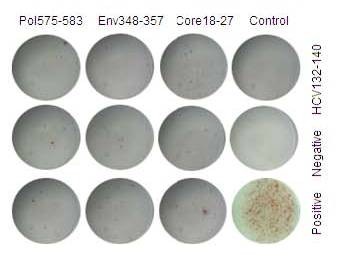
**ELISPOT results from CHB patients**. Sequence of Pol575-58 is FLLSLGIHL, sequence of Env348-357 is GLSPTVWLSV, sequence of Core18-27 is FLPSDFFPSV, sequence of HCV Core132-140 is DLMGYIPLV; all are HLA-A0201 limited. Medium without stimulation or cells was used for negative control, PHA for positive control. 2 × 10E5 cells per well was used for the negative and peptide stimulated groups, 1 × 10E4 cells per well was used for the positive control. The final concentration of peptide was 10 μg/ml, PHA 4 μg/ml. HLA-A0201 positive patients were chosen as the research subjects.

**Figure 5:**
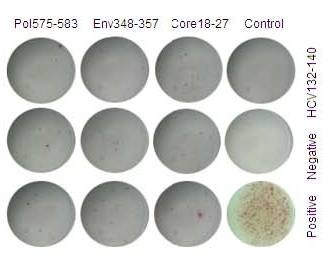
**ELISPOT results from AHB patients**. Sequence of Pol575-58 is FLLSLGIHL, sequence of Env348-357 is GLSPTVWLSV, sequence of Core18-27 is FLPSDFFPSV, sequence of HCV Core132-140 is DLMGYIPLV; all are HLA-A0201 limited. Medium without stimulation or cells was used for negative control, PHA for positive control. 2 × 10E5 cells per well was used for the negative and peptide stimulated groups, 1 × 10E4 cells per well was used for the positive control. The final concentration of peptide was 10 μg/ml, PHA 4 μg/ml. HLA-A0201 positive patients during the first week of hospitalization were chosen as the research subjects.

Pol575-583, Env348-357, or Core18-27 epitope peptides were used to stimulate peripheral blood mononuclear cells (PBMCs) from HBV-infected patients and ELISPOT was used to detect the INF-γ secreting cells in order to understand the INF-γ and other cytokine-secreting ability of the specific CTLs. Our results indicated that the spot-forming units (SFU) of AHB patients at the first week after admission was 132.8 ± 10.8, 194.8 ± 17.7, and 240.4 ± 31.8 for Pol575-583, Env348-357 or Core18-27 epitope peptides, respectively; SFU for CHB patients were 27.2 ± 5.2, 29.1 ± 5.4, and 37.5 ± 6.0 respectively, t = 30.53, 31.17, and 21.75 respectively (p < 0.01 for all). In the AHB group, specific CTLs had a different response to different epitope peptides: Pol575-583<Env348-357<Core18-27, and t = 10.36 for Pol575-583 and Env348-357; 11.10 for Pol575-583 and Core18-27; -4.35 for Env348-357 and Core18-27 (p < 0.01 for each comparison). Stimulation with non-specific HLA-A0201-limited HCV Core132-140 peptide also led to an increase in SFU with no significant difference between the control group and negative group, t = -0.299, p = 0.77. Examples of this ELISPOT analysis can be found in Figures 4 and 5.

### Relationship between HBV DNA level and the frequency and function of HBV specific CTLs

**Figure 6:**
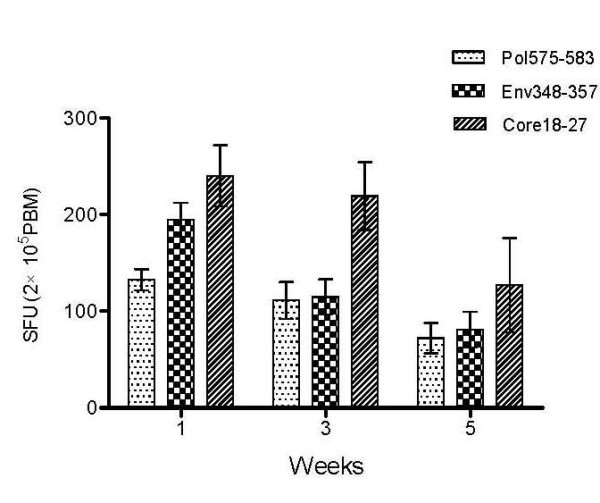
**Dynamic changes of HBV antigen specific CTL responses in AHB patients**. CTL responses against three HBV epitopes (Pol575-583, Env348-357 and Core18-27) were measured by ELISPOT at different time points after admission to hospital.

No statistical differences were observed for the frequencies of specific CTLs in HBV DNA positive and negative AHB patients. However, a follow-up of AHB patients revealed a clearance of serum HBV DNA and normalization of liver function. Furthermore, the HBV-specific CTLs response decreased gradually and was significantly lower than that in the acute phase. This indicated that the potent multiple-epitope CTL responses may be the critical mechanism responsible for the viral clearance (Figure 6).

### Dynamic changes in peripheral blood lymphocyte subsets in AHB patients

**Figure 7:**
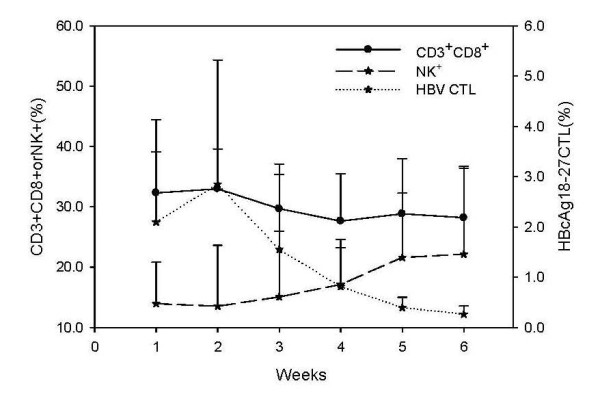
**The relationship between peripheral blood lymphocyte subsets and HBV-specific CTLs**. Dots indicate the mean value. CD3+CD8+ cells and HBcAg18-27-specific CTLs of AHB patients reached peak levels at the second week after admission. There was a positive correlation between them (r = 0.492, p = 0.01). The number of NK cells was negatively correlated with the frequency of the HBV specific CTLs in AHB patients (r = -0.266, p = 0.05).

Numbers of CD3^+^CD8^+ ^cells in AHB patients were significantly higher than those in the healthy control group from the first to the fourth week (for the first week t = 2.766, p = 0.008; second week t = 3.451, p = 0.01). Peak levels of CD3^+^CD8^+ ^cells and the frequency of HBcAg18-27-specific CTLs in AHB patients occurred in the second week after admission. The number of CD3+CD8+ cells was positively correlated with the mean frequency of CTLs at each observation point (r = 0.492, p = 0.01), showing a certain relationship of growth and decline between them (Figure 7).

**Figure 8:**
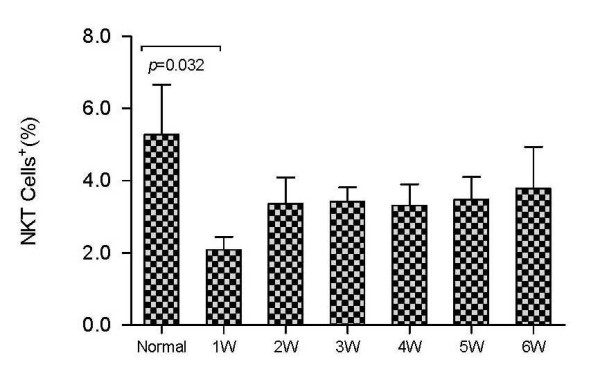
**Levels of NKT cells in either healthy control group (Normal) or AHB patients at different weeks (1 W - 6 W) after being admitted to hospital**.

We also found that although serum HBV DNA and ALT stayed at a high level and NK and NKT cells were relatively low for AHB patients at the first and the second week after admission, NK and NKT cells gradually increased to the normal range with the clearance of the virus and the normalization of liver function. The number of NK cells was negatively correlated with the frequency of HBcAg-specific CTLs during the six weeks of hospitalization (r = -0.266, p = 0.05). NKT cell numbers were lower in AHB patients relative to healthy controls (p = 0.032) in the first week of admission (Figure 8). NKT cells remained low in the following weeks but the difference was not statistically significant.

## Discussion

HBV is a double-stranded DNA virus that can cause acute or chronic liver infection in humans. The antigen cellular immune response plays an important role in the pathogenesis of HBV infection and the clearance of HBV, as has been emphasized in the literature [7].

HBV-specific CD8^+ ^CTLs can kill the hepatitis B virus via cytolytic and non-cytolytic pathways, but they are also the main effector cells for liver injury. In acute HBV infection, HLA class I-restricted CTLs respond specifically to antigen epitopes of the viral membrane, capsid and polymerase proteins; thus, multi-epitope- and multi-clone-specific CD8^+ ^CTLs can be detected. On the other hand, the number of specific CD8^+ ^CTLs in the periphery in patients with chronic hepatitis B is very small. Nakamoto et al. [8] found that in AHB patients with normal immune function, a substantial number of HBV particles are cleared before the liver cells are injured, but a certain amount of the virus still replicates in liver cells, leading to the induction of cytolytic mechanisms and liver cell injury. Thus, HBV is eventually completely cleared, although short-term liver injury can be seen, which is clinically manifested as self-limited AHB. Guidotti et al. [9] studied two chimpanzee models with HBV infection and found that HBV DNA in the serum and liver disappears before the peak in serum transaminases. In the current study, we found that HBcAg-specific CTLs can be detected in the peripheral blood of AHB patients even in the early stages, and their frequency changes dynamically. During hospitalization, HBV CTLs frequencies in AHB patients were significantly higher than those in CHB patients and healthy controls. When serum ALT levels were high, HBV DNA levels decreased significantly. Among AHB patients, 26.4% had negative serum HBV DNA before admission; 50.9% had negative serum HBeAg; and 17.0% had negative serum HBsAg. These findings suggest that serum HBV levels were suppressed or partially cleared before liver transaminase levels became elevated. Meanwhile, peak levels of peripheral blood epitope-specific CTLs in AHB patients occurred in the second week after admission, when the ALT levels had significantly decreased. Peak levels of HBV epitope-specific CTLs appeared later than serum ALT peak levels and lagged behind serum HBsAg and HBV DNA peak levels; furthermore, the higher the frequency of peripheral blood HBV-specific CTLs, the earlier of the antigenic diversion of HBeAg, suggesting that the high frequency of specific CTLs in AHB patients may have played an important role in clearance of the virus. However, the frequency of specific CTLs in AHB patients was not synchronous with liver cell injury or serum HBV clearance, in other words, clearance of the virus occurred earlier than liver cell injury, suggesting that not only cytotoxic effects but also non-cytotoxic effects might be involved in the virus clearance of AHB patients.

Our study indicates that not only was the frequency of specific CTLs significantly higher in AHB patients than in CHB patients, but also the response ability of HBV antigen epitope-specific CD8+ was higher in AHB patients (p < 0.05). The ability of different antigens to stimulate specific CTL to produce INF-γ was different; the pattern observed was as follows: Core18-27> Env348-357> Pol575-583, which may be related to the different immunogenicity of the different regions of HBV antigens [10]. The level of HBV Core18-27-specific response was significantly higher in the acute phase in AHB patients compared with those in convalescence (p < 0.05), which proved that this antigen epitope might be the main target site for host immune clearance of virus [11].

In addition to the characteristics of the specific cell-mediated immune response observed in this study, the non-specific cellular immune response had its own features. The total numbers of peripheral blood cytotoxic T lymphocytes (CD3^+^CD8^+^) in AHB patients in the early stage of infection were higher than in the healthy control group and among CHB patients. The peak of CD3^+^CD8^+ ^cell numbers, meanwhile, appeared at the second week after admission, and the dynamic changes of this cell number were basically the same as that of HBcAg-specific CTL. A correlation was observed between the growth and decline of CD3^+^CD8^+ ^cell numbers and HBcAg-specific CTL (r = 0.492, p = 0.01). These results suggest that the number of peripheral blood CD8 ^+ ^lymphocytes might reflect CTLs frequency indirectly in AHB patients and was helpful to predict the prognosis of an HBV infection.

NK cells are the first line of host defense against the foreign invasion of pathogenic microorganisms [12]. They are usually activated in the early stage of a viral infection and are particularly abundant in the liver [13,14]. Activated NK cells play an important role in the recirculation of virus-specific T cells and the induction of anti-viral immunity in the liver [15]. Previously, we compared lymphocytes in the liver and peripheral blood of patients with different types of hepatitis and found that NK cells in the liver were significantly more abundant than in the peripheral blood [16]. This tendency shows that NK cells play an important role in local immunity in the liver. Our study analyzed peripheral blood NK cells in AHB patients and found that dynamic changes in peripheral blood NK cells were different from the trend of the frequency of HBcAg-specific CTLs. The NK cells in these patients gradually increased to the normal range as the virus was cleared in the peripheral blood and liver function recovered and the dynamic changes of peripheral blood NK cells negatively correlated with the frequency of HBV-specific CTL (r = -0.266, p = 0.05). These results suggest that NK cells play an extremely important role in the effective clearance of HBV in the early stage of infection.

NKT cells are highly heterogeneous effector cells with immune and regulatory functions [17]. They possess the characteristics of both NK cells (CD56^+^) and T lymphocytes (CD3^+^) [18]. NKT cells are very rich in the mouse liver and account for 50% of intrahepatic lymphocytes, mainly located at the hepatic sinusoids [19]. They play an important role in the prevention of tumor metastasis and the removal of viruses [20]. It was shown, by a mouse hepatitis model induced by Con A or LPS plus IL-12, that NKT cells could induce liver damage, and the activated NKT cells disappeared from liver [21,22]. The activation of NKT cells is often accompanied by the activation of NK cells, which then release cytokines that prevent viral replication [23]. Therefore, it is thought that NK cells might be effector cells for NKT cells [24]. In the healthy control group, 2% of the CD3^+ ^cells in the peripheral blood expressed CD56, whereas in the liver, this value was 30% [25,26]. In our study, the number of NKT cells in the peripheral blood of AHB patients in the early stage of infection was significantly lower than in the control group, and the dynamic changes in NKT cells were basically the same as those in NK cells. Based on these findings, we suggest that NK and NKT cells accumulate in the liver and play an important role in local immunity, resulting in a lower frequency of NKT cells in the peripheral blood.

## Conclusions

In summary, activated NK and NKT cells are involved in the non-specific removal of virus at an early stage of infection, particularly for viral clearance in liver cells [27,28]. Specific cellular immunity induced by HBV infection plays a key role in the total clearance of HBV. During dynamic monitoring of the function and number of specific CTL, NK, and NKT cells during acute HBV infection, we found that changes had great clinical significance in determining the prognosis of HBV infection and in evaluating the chronic process.

## Competing interests

The authors declare that they have no competing interests.

## Authors' contributions

JL, YH, SL, SW, and ZH participated in the study design and paper discussion; JL and YH wrote the paper; SL edited manuscript; YH, KJ, and YW participated in the specimen collection and testing; BL and YL participated in the data analysis; all authors read and approved the final manuscript.
